# Retinal iron homeostasis in health and disease

**DOI:** 10.3389/fnagi.2013.00024

**Published:** 2013-06-28

**Authors:** Delu Song, Joshua L. Dunaief

**Affiliations:** The F.M. Kirby Center for Molecular Ophthalmology, Scheie Eye Institute, Perelman School of Medicine at University of PennsylvaniaPhiladelphia, PA, USA

**Keywords:** iron, retina, age-related macular degeneration (AMD), chelator, oxidative stress, ferroportin, ceruloplasmin, hephaestin

## Abstract

Iron is essential for life, but excess iron can be toxic. As a potent free radical creator, iron generates hydroxyl radicals leading to significant oxidative stress. Since iron is not excreted from the body, it accumulates with age in tissues, including the retina, predisposing to age-related oxidative insult. Both hereditary and acquired retinal diseases are associated with increased iron levels. For example, retinal degenerations have been found in hereditary iron overload disorders, like aceruloplasminemia, Friedreich's ataxia, and pantothenate kinase-associated neurodegeneration. Similarly, mice with targeted mutation of the iron exporter ceruloplasmin and its homolog hephaestin showed age-related retinal iron accumulation and retinal degeneration with features resembling human age-related macular degeneration (AMD). *Post mortem* AMD eyes have increased levels of iron in retina compared to age-matched healthy donors. Iron accumulation in AMD is likely to result, in part, from inflammation, hypoxia, and oxidative stress, all of which can cause iron dysregulation. Fortunately, it has been demonstrated by *in vitro* and *in vivo* studies that iron in the retinal pigment epithelium (RPE) and retina is chelatable. Iron chelation protects photoreceptors and retinal pigment epithelial cells (RPE) in a variety of mouse models. This has therapeutic potential for diminishing iron-induced oxidative damage to prevent or treat AMD.

## Introduction

As an important part of many metabolic processes, iron is essential for life. However, excess iron can be toxic to tissues. Iron is a crucial component of enzymes of the citric acid cycle and electron transport chain, making iron essential for adenosine triphosphate (ATP) production (Wigglesworth and Baum, 1988; Poss and Tonegawa, 1997). Iron is also required by ribonucleoside reductase which is the rate-limiting enzyme of the first metabolic reaction in DNA synthesis (Wigglesworth and Baum, 1988). In the central nervous system (CNS), oligodendrocytes require iron for myelin synthesis and maintenance (LeVine and Macklin, 1990; Morris et al., 1992). Iron is an essential cofactor for synthesis of neurotransmitters, including dopamine, norepinephrine, and serotonin (Youdim, 1990). Thus, disruption of iron homeostasis may be involved in diseases of the CNS. Iron deficiency in children can cause auditory defects due to myelin disruption (Roncagliolo et al., 1998). Demyelinating diseases like multiple sclerosis are associated with cellular iron homeostasis dysfunction (Drayer et al., 1987).

In retina, iron is particularly critical for the visual phototransduction cascade. RPE65, an iron containing protein, is dependent on iron for isomerohydrolase activity. In the retinal pigment epithelium (RPE), this activity is important in catalyzing the conversion of all-trans-retinyl ester to 11-cis-retinol in the visual cycle (Moiseyev et al., 2005). 11-cis-retinol leads to 11-cis-retinaldehyde, the photosensitive component of rhodopsin. Photoreceptor cells continuously shed and synthesize their disc membranes, depending on iron-containing enzymes like fatty acid desaturase for biogenesis of lipids which is used in disc membrane replacement (Shichi, 1969). Although iron is essential for retinal function, excessive iron can be toxic. Ferrous iron can catalyze the conversion of hydrogen peroxide to hydroxyl radical, which is the most damaging of the reactive oxygen species. Oxidative damage caused by hydroxyl radical includes lipid peroxidation, DNA strand breakage and biomolecule degradation (Halliwell and Gutteridge, 1984). Hydroxyl radicals have been implicated in the pathogenesis of Alzheimer's and other CNS diseases (Smith et al., 1997). Since the main function of the RPE cells is phagocytize lipid-rich, easily oxidized photoreceptor outer segments in a high oxygen tension environment, the retina and RPE are particularly prone to oxidative damage. Therefore, iron must be carefully regulated in the eye to provide necessary iron without giving rise to oxidative damage. Retinal iron deficiency has not been found to be a clinical problem, perhaps because the retina is very efficient at taking up whatever iron is available in the circulation, and/or because retinal iron stores significantly exceed iron needs.

## General iron homeostasis

In the circulation, iron exists in two forms: heme and non-heme iron. The majority of non-heme iron is bound to transferrin (Baker and Morgan, 1994), which is capable of binding two molecules of ferric iron. Adults have approximately 3 mg of circulating non-heme iron, only saturating 30% of transferrin binding sites. However, transferrin cannot cross the tight junctions of the blood brain barrier (BBB); therefore, cells that comprise the BBB must import iron and transfer it into neural tissue. Iron-laden transferrin binds to transferrin receptor (TfR) on the cell surface, and this complex is internalized into endosomes. In low pH, iron is released (Sipe and Murphy, 1991) and exported by endosomes for use, storage in ferritin, a multisubunit protein consisting of H- and L-chains (Aisen et al., 2001) or export. Upon iron delivery, transferrin is recycled to the surface (Hunt and Davis, 1992).

Ferroportin exports ferrous iron that is not utilized or stored in the cell (Abboud and Haile, 2000; Donovan et al., 2000; McKie et al., 2000), but exported iron must be oxidized to be accepted by transferrin in the circulation. Oxidation of iron is accomplished by the ferroxidases such as, ceruloplasmin (Osaki et al., 1966), hephaestin (Heph) (Vulpe et al., 1999), amyloid precursor protein (APP) (Duce et al., 2010) or zyklopen (Chen et al., 2010). These enzymes are crucial for iron export and when disrupted result in cellular iron accumulation and degeneration. Ceruloplasmin, a copper binding protein, contains over 95% of copper found in plasma. Mutation of a copper transporter in Wilson's disease causes decreased serum ceruloplasmin levels resulting from failure of copper incorporation into ceruloplasmin. Hephaestin is 50% identical to ceruloplasmin at the amino acid level. In the sex-linked anemia mouse, *heph* has been identified as the disrupted gene product. In these mice, failure of hephaestin to export iron from the enterocyte into the circulation results in low serum iron and enterocyte iron increase (Vulpe et al., 1999). Unlike ceruloplasmin, which is a secreted plasma protein or glycosylphosphatidylinositol (GPI) membrane-anchored protein (Patel and David, 1997), Heph is present only as a membrane-bound protein. APP, a type I transmembrane protein precursor, was found to be a functional ferroxidase. Both full-length and soluble APP species can interact with ferroportin to facilitate iron export from neurons and other cells (Duce et al., 2010). In Alzheimer's disease, iron export by APP is inhibited by elevated extracullular Zn^2+^ dissociating from β-Amyloid. The APP domain responsible for ferroxidase activity has been debated (Ebrahimi et al., 2012). In 2010, zyklopen was identified as another multicopper ferroxidase and plays a role in placental iron transport. Immunostaining of zyklopen identified its expression in the brain, kidney, testes and retina but not in the liver or intestine (Chen et al., 2010). Our unpublished data also suggests that zyklopen is expressed in brain and neural retina, but not RPE cells.

Iron levels are managed by an iron-responsive mechanism involving iron regulatory proteins (IRPs, IRP1 and IRP2) which serve to register intracellular iron status. This regulation allows individual cells to regulate iron uptake, sequestration and export depending on their iron status. Under conditions of iron starvation, IRP1 and IRP2 are activated for high affinity binding to multiple “iron-responsive elements” (IREs) in the 3'-untranslated region of TfR mRNA and to a single IRE in the 5'-untranslated region of the mRNAs encoding both H- and L-ferritin chains. This stabilizes TfR mRNA (Binder et al., 1994) and inhibits ferritin mRNA translation (Muckenthaler et al., 1998). Conversely, failure of IRPs to bind to cognate IREs in iron-replete cells leads to degradation of TfR mRNA and synthesis of ferritin.

## Iron import into the retina

The blood–retinal barrier isolates the retina from the bloodstream. The intercellular tight junctions of the neuroretinal vasculature and RPE resulting in independent barriers prevent intercellular diffusion, thereby protecting both sides of the retina from systemic circulation.

### Transferrin mediated transport

When bound to transferrin in the choroidal circulation, iron is taken up by high affinity TfR at the basolateral surface of the RPE. Then, this complex is internalized into a low pH endosome where iron is dissociated from transferrin. After export from the endosome, iron enters a cytoplasmic pool of labile iron where it can bind to ferritin or be transported for further use. Whether RPE cells transport iron apically toward the retina is not clear. A potential alternative route for iron transport into the retina is through the retinal vascular endothelial cells. Evidence suggests that ferroportin is expressed by these cells, which may transport iron into the retina in a process regulated by hepcidin (Hadziahmetovic et al., 2011a). Once iron enters the retina, it is likely to bind transferrin. The vitreous humor and aqueous fluid contain large amounts of transferrin (Hawkins, 1986; Yu and Okamura, 1988; Tripathi et al., 1990). Experiments with intravitreal injection of labeled fucose or tyrosine suggest that the vitreous transferrin is partially synthesized locally in the eye (Laicine and Haddad, 1994), in part by the ciliary body (Rodrigues et al., 1998). The iron-transferrin complex can then be taken up by the TfRs on the photoreceptor inner segments (Yefimova et al., 2000). The photoreceptor inner segments of adult rat retinas are immunolabled for TfR, which probably bind and internalize the iron-transferrin complex in the photoreceptor matrix.

### Divalent metal transporter-1

Divalent metal transporter-1 (DMT1), a proton symporter, moves one atom of ferrous iron and a proton in the same direction (Gunshin et al., 1997). DMT1 is located on the apical surface of intestinal epithelial cells and transports dietary free iron, upon reduction from the ferric to ferrous form, from the luminal surface into the enterocytes (Rouault and Cooperman, 2006). DMT1 is located on the endosome and transports iron from the endosome into the cytoplasm in most other cells in the body (Rouault and Cooperman, 2006). Some studies have localized DMT1 in rat brain blood vessels (Burdo et al., 2001), while others have detected it in neurons but not vascular cells (Gunshin et al., 1997). While this issue remains controversial, its potential localization in blood vessel suggests that DMT1 may mediate iron transport to and/or from the brain. Belgrade rats with a mutation in DMT1 have a hypochromic, microcytic anemia and less iron in their brains, consistent with a role for DMT1 in brain iron transport (Burdo et al., 1999). In the retina, we detected strong DMT1 immunolabel in rod bipolar cell bodies, rod bipolar cell axons, horizontal cell bodies, and photoreceptor inner segments. However, the importance of DMT1 for iron import into these cells is currently unclear.

### Dexras

Dexras1, a 30 kDa G-protein with close homology to the Ras subfamily and localized predominantly to the brain, can be induced by the activation of glutamate-NMDA receptors to signal the uptake of iron in neurons (Cheah et al., 2006). The activation of glutamate-NMDA receptors stimulates nitric oxide synthase which binds to the nitric oxide synthase 1 adapter protein, CAPON, and delivers nitric oxide to Dexras1 causing S-nitrosylation of Dexras1. In the down-stream pathway, Dexras1 interacts with peripheral benzodiazepine receptor-associated protein (PAP7), which binds to DMT1 to induce iron uptake. It has been hypothesized that NMDA neurotoxicity is caused by increased iron uptake following induction of this signaling cascade, since iron chelation in neuronal cultures protects against NMDA neurotoxicity. Consistent with this mechanism, Dexras1 knockout mice are protected against retinal ganglion cell death induced by intravitreal NMDA injection (Chen et al., 2013).

## Iron storage

### Ferritin

Iron is primarily stored as cytosolic ferritin which is capable of holding as many as 4500 iron molecules in the ferric state in its central core. H-ferritin has ferroxidase activity allowing it to rapidly accumulate Fe^3+^ in its central core (Aisen et al., 2001) while L-ferritin does not (Levi et al., 1994). The distribution of iron and ferritin has been characterized in the adult rat retina. Proton-induced X-ray emission identified the largest amounts of heme and non-heme iron in the inner segments of photoreceptors, the RPE, the choroid, the inner nuclear layer, and the ganglion cell layer (Yefimova et al., 2000). Iron was present in somewhat lesser, but still significant amounts in the photoreceptor outer segments (Yefimova et al., 2000; Ugarte et al., 2012). Interestingly, immunohistochemistry studies have demonstrated a similar distribution pattern of retinal ferritin. The exception is that iron, but not much ferritin, is contained in the photoreceptor outer segment. Also, ferritin levels are regulated by IRPs (Hentze and Kühn, 1996). IRPs inhibit ferritin translation by binding to ferritin mRNA in iron-depleted cells to regulate ferritin levels.

### Mitochondrial ferritin

Mitochondrial ferritin (TfR) is structurally similar to H-ferritin but localized to the mitochondria (Levi et al., 2001). MtF was detected by immunohistochemistry in the photoreceptor inner segments and diffusely throughout the inner retina (Hahn et al., 2004b). In order to confirm mitochondrial localization, anti-MtF was co-labeled with an antibody specific for the ATPase in Complex V of the electron transport chain of mitochondria. Label co-localized to the inner segment ellipsoids, the location of the mitochondria, but not the inner segment myoid.

## Iron export

### Ceruloplasmin

Ceruloplasmin, a multicopper oxidase with ferroxidase activity, functions as an antioxidant by oxidizing iron from its Fe^2+^ to Fe^3+^ state, thus preventing oxidative damage induced by Fe^2+^ mediated free radical generation by the Fenton Reaction (Osaki, 1966). Additionally, by oxidizing iron from its Fe^2+^ to Fe^3+^, ceruloplasmin facilitates iron export out of the cell since only ferric iron can be taken up by transferrin in the circulation (Osaki, 1966); extracellular ferroxidases including ceruloplasmin are important in the ferroxidation which is thus necessary to initiate efficient iron export, probably through the generation of an ion gradient (Sarkar et al., 2003).

Two forms of ceruloplasmin have been identified: the secreted form and the membrane-anchored GPI-linked form. The secreted form is the predominant form made by the liver while the GPI-linked form is the predominant form found in the brain (Patel et al., 2000). Both forms of ceruloplasmin are present in mouse and human retina (Chen et al., 2003). The secreted form was also identified in the mouse and human RPE (Hahn et al., 2004b). Immunohistochemistry demonstrated that ceruloplasmin protein is located diffusely throughout the retina. The strongest signal in mouse retina was found in the Müller cells. As confirmed by Western analysis, ceruloplasmin is also present in the aqueous, vitreous and retina of a normal human eye (Chen et al., 2003).

Ceruloplasmin has been found to be upregulated in the retina in multiple pathological conditions including light damage, optic nerve crush, glaucoma, and diabetes (Levin and Geszvain, 1998; Chen et al., 2003; Miyahara et al., 2003; Farkas et al., 2004; Gerhardinger et al., 2005). In 10-week-old male BALB/c mice exposed to bright fluorescent light or room light for 7 h, retinal ceruloplasmin levels were increased in light exposed retinas compared to controls. Again, the strongest label was observed in the Müller cells (Chen et al., 2003). It was postulated that upregulation of ceruloplasmin following light damage serves to protect the eye from light-induced oxidative attack. Both the direct antioxidant effect of ceruloplasmin as well as its iron export function may be important for protection against light damage.

In rats subjected to intraorbital optic nerve crush, ceruloplasmin mRNA levels were found to be upregulated relative to controls. Ceruloplasmin mRNA localized to the inner nuclear and ganglion cell layers demonstrated by *in situ* hybridization. Immunohistochemistry localized ceruloplasmin protein in sporadic cells within the nerve fiber layer of untreated retinas (Levin and Geszvain, 1998). In monkeys with experimental glaucoma, microarray assay has demonstrated ceruloplasmin upregulation and immunohistochemical studies have localized the ceruloplasmin to Müller cells (Miyahara et al., 2003). In human retinas with glaucoma, there are increased ceruloplasmin levels in the inner plexiform layer, ganglion cell layer, and nerve fiber layer compared with controls by immunohistochemistry (Farkas et al., 2004).

In addition, ceruloplasmin has also been shown to be upregulated in the diabetic rat retina (Gerhardinger et al., 2005). Similarly, ceruloplasmin is upregulated in the aqueous and vitreous of rabbit eyes with endotoxin-induced ocular inflammation (McGahan and Fleisher, 1986). Ceruloplasmin is additionally upregulated secondary to oxidative stress to the lens, as confirmed by exposure of immortal murine lens epithelial cells to the oxidant *tert*-butyl hydroperoxide (Li et al., 2004).

### Hephaestin

Hephaestin (Heph), another multicopper ferroxidase, is a homolog of ceruloplasmin with 50% amino acid identity (Vulpe et al., 1999). Similar to ceruloplasmin, Heph facilitates iron export by oxidizing ferrous iron to its ferric state thereby facilitating its transport across the plasma membrane and uptake by transferrin in the extracellular space (Vulpe et al., 1999). Vulpe et al identified Heph through characterization of sex-linked anemia (sla) mouse which has a Heph mutation that severely decreases its ferroxidase activity. In this mouse, decreased Heph ferroxidase activity resulted in reduced iron export from intestinal epithelial cells, yielding an anemic mouse.

**Figure 1:**
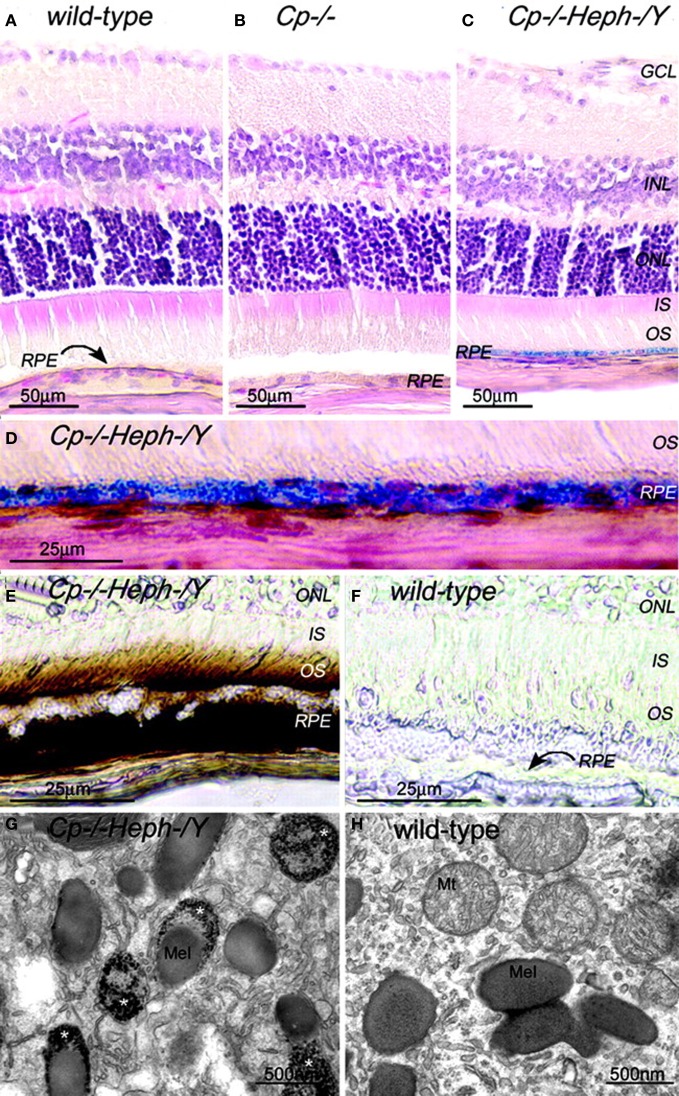
**Adult (6-month-old) Cp^−/−^Heph^−^/Y RPE and photoreceptors accumulate iron. (A–C)** 6-month-old WT **(A)**, Cp^−/−^ **(B)**, and Cp^−/−^Heph^−^/Y **(C)** retinas Perls' stained for iron (blue) and counterstained with hematoxylin/eosin. **(D)** High magnification of Prussian blue Perls' label in 6-month-old Cp^−/−^Heph^−^/Y RPE. (**E** and **F**) Light photomicrographs of 6-month-old Cp^−/−^Heph^−^/Y **(E)** and WT **(F)** retinas after DAB enhancement (brown) of Perls' stain. (**G** and **H**) Electron micrographs of RPE from 6-month-old Cp^−/−^Heph^−^/Y **(G)** and WT **(H)** eyes. Only the Cp^−/−^Heph^−^/Y RPE **(G)** contains electrondense vesicles (^*^) sometimes fused with melanosomes. Reprinted from Hahn et al., (2004b).

The presence of *heph* mRNA has been confirmed by RT-PCR in mouse and human RPE cells, and the localization of Heph protein also has been demonstrated by immunohistochemistry in Müller cells in the retina with the greatest amounts in the Müller endfeet adjacent to the internal limiting membrane (Hahn et al., 2004b). The importance of ceruloplasmin and Heph in iron export from the retina is demonstrated in mice with combined deficiency of these two enzymes that results in age-dependent iron accumulation in both RPE and retina (Hahn et al., 2004b). In these mice, Perls' staining revealed iron accumulation in the photoreceptor outer segments and RPE, and electron micrographs showed electron-dense vesicles in the RPE (Figure 1). These vesicles were likely lysosomes or endosomes, sometimes fused with melanosomes. X-ray spectroscopy demonstrated that these vesicles had four times more iron content compared to other intracellular structures. Immunohistochemistry revealed H- and L-ferritin were present in rod bipolar cell termini in the inner plexiform layer. Additionally, L-ferritin was found in the inner sections of the outer plexiform layer and the RPE. H-ferritin label showed strongest signal in the photoreceptor inner segments and axons in the outer part of the outer plexiform layer (Hahn et al., 2004a). In contrast to cytosolic H- and L-ferritin, MtF has not been shown to be IRP-regulated. But MtF was increased in the photoreceptor inner segments of Cp^−/−^ and Cp^−/−^Heph^−^/Y mice (Hahn et al., 2004a). In Cp^−/−^mice, MtF colocalized with a mitochondria-specific antibody to the inner segment ellipsoid rather than the inner segment myoid. The elevated iron level in these mice causes oxidative stress (detected by increased isoprostane levels) resulting in retinal degeneration characterized by RPE hypertrophy and autofluorescence, sub-RPE deposits, photoreceptor death, and subretinal neovascularization. This degeneration is prevented by the oral iron chelator deferiprone (Hadziahmetovic et al., 2011b).

### Ferroportin and hepcidin

Evidence suggests that Ferroportin (Fpn), a transmembrane protein, exports iron out of the cell in cooperation with ferroxidases Cp or Heph which convert it to ferric form (Harris et al., 1999; Vulpe et al., 1999). Ceruloplasmin increased iron export from *Xenopus* oocytes in the presence of Fpn (Donovan et al., 2000; McKie et al., 2000). This effect was observed when ceruloplasmin was added to the medium (McKie et al., 2000). Ceruloplasmin also exports iron from macrophages *in vitro*, but only when tissue culture oxygen levels mimick *in vivo* tissue oxygen levels rather than atmospheric oxygen levels (Sarkar et al., 2003). Furthermore, stable expression of Fpn in J774 cells, a macrophage cell line, increases iron efflux after erythrophagocytosis (Knutson et al., 2005). Conditional knockout of Fpn in mouse villus enterocytes has shown that the protein functions as the major, if not only, iron exporter from the duodenum to the circulation (Donovan et al., 2005).

In the mouse retina, ferroportin immunolocalizes to the RPE, photoreceptor inner segments, inner and outer plexiform layers, and ganglion cell layer. In mice deficient in Cp and Heph, ferroportin immunoreactivity increases, presumably due to an iron-mediated increase in levels of ferroportin transcription (Hahn et al., 2004a).

Hepcidin (Hepc) is a 25 amino acid peptide found in human plasma and urine and produced by the liver (Krause et al., 2000; Park et al., 2001). It was shown that Hepc has anti-microbial activity, and its expression is upregulated by infection, inflammation and iron (Pigeon et al., 2001; Nicolas et al., 2002). Hepc binds to ferroportin, triggering its internalization and lysosomal-dependent degradation (Nemeth et al., 2004; Knutson et al., 2005). Liver secretes Hepc into the circulation when iron is in excess. The Hepc then triggers degradation of ferroportin in enterocytes and macrophages, maintaining homeostasis by preventing iron release into the circulation. In the retina, Hepc can also be synthesized by Muller cells, photoreceptors and RPE and is upregulated by inflammation (Gnana-Prakasam et al., 2008) and elevated iron levels (Hadziahmetovic et al., 2011b). Ferroportin degradation in vascular endothelial cells and RPE is triggered by Hepc, preventing further iron transport into the neural retina, which is consistent with Hepc knockout mice exhibiting increased retinal iron and subsequent retinal degeneration (Hadziahmetovic et al., 2011b). Bmp6 is crucial for regulation of systemic iron homeostasis through Hepc. Bmp6 was detected in RPE and its receptors are expressed in the neurosensory retina. In cultured RPE cells, Bmp6 was down-regulated by oxidative stress and up-regulated by iron. Bmp6 secreted from the RPE cells likely binds Bmp6 receptors in the neurosensory retina up-regulating Hepc, because both intravitreal and subretinal injection of Bmp6 up-regulated Hepc within the neurosensory retina but not within the RPE. Bmp6^−/−^ mice had age-dependent retinal iron accumulation and degeneration. *Postmortem* RPE from patients with early AMD exhibited decreased Bmp6 levels. The diminished Bmp6 levels observed in RPE cells in early AMD may contribute to iron build-up in AMD. This may in turn propagate a vicious cycle of oxidative stress and iron accumulation, exacerbating AMD and other diseases with hereditary or acquired iron excess (Hadziahmetovic et al., 2011c).

## Disruption of iron homeostasis and oxidative damage

Increased intraocular iron has been shown to cause oxidative injury to the retina as shown when intravitreous iron sulfate is administered to adult C57BL/6 mice, resulting in increased superoxide radicals in photoreceptor inner segments, lipid peroxidation of the photoreceptors and retinal degeneration (Rogers et al., 2007). With a higher concentration of iron sulfate injection, these mice exhibited an irregular outer border of the outer nuclear layer suggesting photoreceptor damage. Ceruloplasmin-deficient mice have hereditary iron overload and also show features of retinal degeneration. Eighteen-month-old Cp^−/−^ mouse retinas showed mild degeneration of the inner nuclear layer including condensed chromatin and dark cytoplasm in the cells (Patel et al., 2002). Patients with ceruloplasmin deficiency from the hereditary disease aceruloplasminemia also have retinal iron accumulation with retinal degeneration (Dunaief et al., 2005). The degeneration primarily involves RPE cells, with hypo and hyperpigmentation, autofluorescence, sub-RPE drusen and subretinal drusenoid deposits (Wolkow et al., 2011).

**Figure 2:**
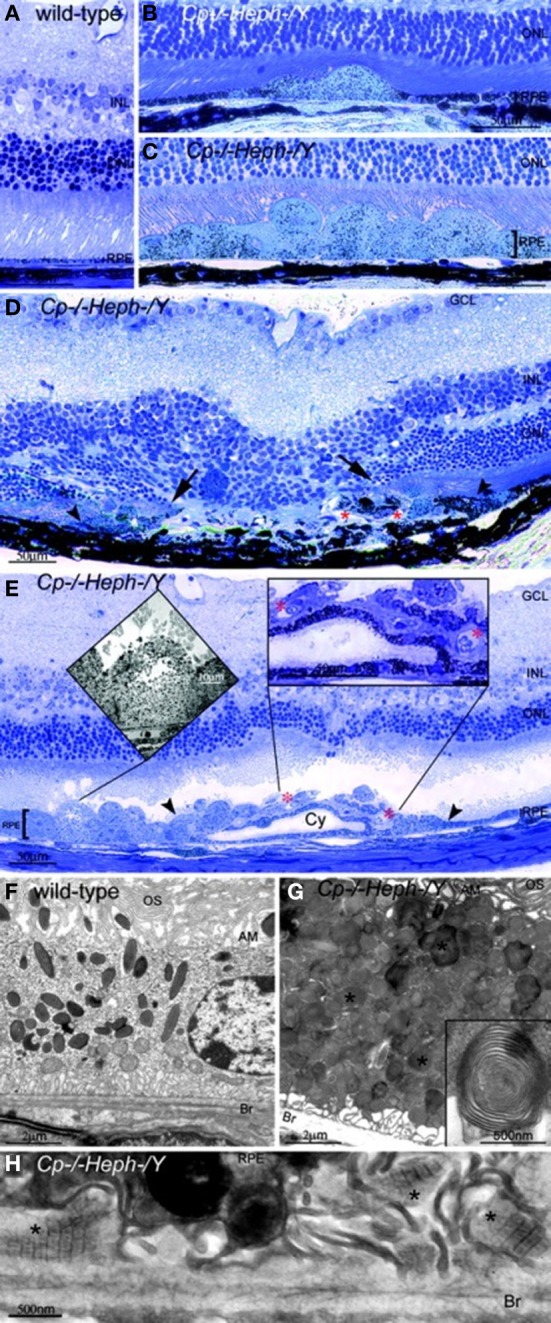
**Cp^−/−^Heph^−^/Y (9-mon-old) mice have retinal degeneration. (A)** Light photomicrograph of WT retina. (**B** and **C**) Cp^−/−^Heph^−^/Y retina has focal patches of hypertrophic RPE cells in some regions **(B)** and confluent hypertrophic RPE cells in other areas **(C)**. **(D)** In an area of RPE hyperplasia (demarcated by arrowheads), Cp^−/−^Heph^−^/Y retinas have local photoreceptor degeneration [demarcated by arrows in the outer nuclear layer (ONL)] and subretinal neovascularization (red^*^). **(E)** In an area of hypertrophic, hyperplastic (area demarcated by arrowheads) RPE cells, a necrotic RPE cell also observed by electron microscopy (Left Inset) is present. Within the area of RPE hyperplasia, there is local photoreceptor thinning and subretinal neovascularization (red^*^) visible as small vessels containing erythrocytes (Right Inset). The hyperplastic RPE have formed a localized cyst (Cy). **(F)** Electron micrograph of WT RPE. Br, Bruch's membrane; AM, apical microvilli; OS, photoreceptor outer segments. **(G)** Electron micrograph of Cp^−/−^Heph^−^/Y RPE overloaded with phagosomes and lysosomes containing photoreceptor outer segments at various stages of digestion. Some of these lysosomes (^*^) contained multilamellar structures characteristic of outer segment membranes (Inset). **(H)** Electron micrograph of Cp^−/−^Heph^−^/Y deposits between RPE and Bruch's membrane containing wide-spaced collagen (^*^). (Scale bars: **A–E**, 50 μm; **F** and **G**, 2 μm; **H**, 500 nm.) Reprinted from Hahn et al., (2004b).

Our team has found that mice with age-dependent iron accumulation from combined deficiency of Cp and Heph also exhibit retinal degeneration (Hahn et al., 2004b). In these mice, RPE cells in up to 75% of a histologic section across the entire retina were severely hypertrophic, hyperplastic, and necrotic with local photoreceptor loss and subretinal neovascularization revealed by light microscopy. Electron microscopy of the hypertrophic RPE revealed excessive accumulation of phagosomes and lysosomes which appear to contain undigested outer segments (Figure 2).

Disrupted RPE-photoreceptor interactions can cause iron overload that probably exacerbates retinal degeneration (Yefimova et al., 2002). In Royal College of Surgeons (RCS) rats with a mutation in the receptor tyrosine kinase Mertk, the RPE is unable to phagocytise shed outer segments, resulting in a layer of undigested outer segment tips in the subretinal space (Yefimova et al., 2002). This disrupts the normal function of RPE-photoreceptor interactions, including transferrin diffusion, and leads to increased non-heme iron levels in the interphotoreceptor debris layer, and diminished transferrin levels in the photoreceptor layer, both of which promote photoreceptor loss at *post-natal* day 20.

## Retinal disorders resulting from abnormal retinal iron metabolism

**Figure 3:**
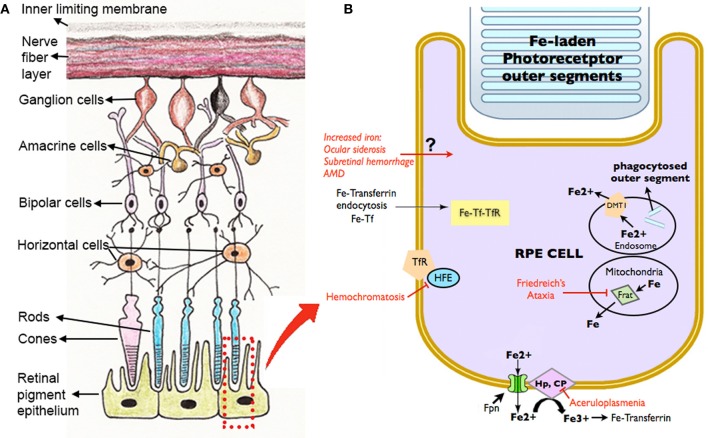
**Schematic diagram showing the retinal layers (A), with enlarged view of the RPE and potential routes of iron traffic (B).** Several diseases that disrupt retinal iron transport are indicated in red. Proteins involved in iron transport are illustrated: Cp, ceruloplasmin; DMT1, Divalent metal tranporter-1; Fe, iron; Fe^2+^, ferrous iron; Fe^3+^, ferric iron; Fpn, ferroportin; Frat, Frataxin; HFE, histocompatability leukocyte antigen class I-like protein involved in iron homeostasis; Hp, Hephaestin; Tf, transferrin; TfR, Transferrin receptor.

Disrupted iron homeostasis in hereditary and acquired diseases can result in iron overload and retinal disease. Putative retinal iron transport mechanisms and several diseases affecting them are summarized in Figure 3.

### Age-related macular degeneration

AMD is the leading cause of irreversible blindness in people over age 65 in developed nations (Leibowitz et al., 1980; Klein et al., 1995). In early stage, manifestations of the disease include drusen deposits associated with the RPE. As the disease progresses, patients may develop geographic atrophy in dry AMD or non-exudative AMD; choroidal neovascularization in wet or exudative AMD. Oxidative stress and radical mediated damage are implicated in the pathogenesis of AMD (Beatty et al., 2000; Zarbin, 2004). In a large clinical trial, patients with intermediate stage AMD given dietary supplements of antioxidants including beta-carotene, vitamin C, vitamin E, and zinc, experienced reduced progression to advanced AMD. This suggests oxidative stress is an important factor involved in AMD pathogenesis (AREDS, 2001).

Iron may be a major source of oxidative stress in AMD. Perls' stain detected increased iron in AMD-affected maculas compared to healthy age-matched maculas (Hahn et al., 2003), specifically in the RPE and Bruch's membrane of early AMD, geographic atrophy, and exudative AMD patients. Analysis of the *postmortem* macula of a 72-year-old white male with advanced geographic atrophy demonstrated iron overload not only in the RPE but also in the photoreceptor layer. Perls' stain detected iron in the photoreceptors and internal limiting membrane along with elevated levels of ferritin and ferroportin in the photoreceptors and internal limiting membrane whereas the normal maculas were only weakly labeled with anti-ferritin and anti-ferroportin antibody (Dentchev et al., 2005). Mean aqueous humor iron levels were elevated by more than two-fold in dry AMD patients undergoing cataract surgery relative to controls, supporting the assertion that iron levels are elevated in AMD eyes (Jünemann et al., 2013).

Although iron accumulation has been detected in AMD retinas, whether it is a cause or consequence of AMD remains elusive. However, there are several lines of evidence indicating that iron directly contributes to AMD pathogenesis. First, iron accumulates with age increase (Hahn et al., 2006). Eyes from younger donors (less than 35 years old) and older donors (older than 65 years old) were dissected and separated into the retina and RPE/choroid. Atomic absorption spectrophotometry of retinas showed a significant increase in iron levels in the retinas of older compared to younger eyes. Much of this iron in the neural retina is likely to be in the photoreceptor layer, which is the region containing the most iron in rat retinas (Ugarte et al., 2012).

Second, iron is a potent generator of radicals and results in photoreceptor/RPE toxicity when locally elevated in conditions such as ocular siderosis. Although siderosis causes pan-retinal degeneration but not drusen, geographic atrophy, or CNV, this variation may be attributed to the differences in route of iron delivery and spatial and temporal patterns of iron accumulation. Third, mice with double deficiency of Cp and Heph and subsequent age dependent retinal iron accumulation develop a retinal degeneration with several features of AMD (Hahn et al., 2004b). These features include sub-RPE wide-spaced collagen that is normally found in association with drusen in humans, RPE lysosomal inclusions, and RPE death. Histologically, these mice show focal photoreceptor loss and subretinal neovascularization similar to the advanced stage of AMD (Figure 2). Fourth, a patient with iron overload resulting from aceruloplasminemia had early-onset subretinal deposits similar to drusen found in AMD patients (Dunaief et al., 2005). Finally, transferrin is upregulated in AMD patients suggesting altered iron regulation or levels (Chowers et al., 2006). Microarray analysis and RT-PCR on *postmortem* retinas of AMD patients and age-matched controls found a 3.5-fold increase in transferrin mRNA levels in dry AMD relative to retinas without AMD and 2.1-fold increase in wet AMD vs. retinas without AMD. Western blot analysis showed a 2.1-fold increase in transferrin levels in AMD retinas compared to retinas without AMD. In these AMD patients, immunohistochemistry detected more transferrin signal in AMD retinas, especially in the photoreceptors, Müller cells, and drusen.

While the mechanism of iron accumulation in AMD is unknown, plausible culprits include inflammation, hypoxia, and oxidative stress, all of which contribute to AMD pathogenesis. Inflammation can cause cellular iron sequestration through IL-6 mediated upregulation of hepcidin (Ganz and Nemeth, 2009). Hypoxia can lead to increased iron uptake through HIF-mediated upregulation of DMT1, a cellular iron importer (Wang et al., 2010). Oxidative stress can upregulate TfR, another cellular iron importer (Fonseca et al., 2003). Further, hemorrhage in wet AMD causes iron transfer from degraded red blood cells to the retina (Bhisitkul et al., 2008).

### Aceruloplasminemia

Aceruloplasminemia is a rare adult-onset autosomal recessive disease caused by mutations in the ceruloplasmin gene on chromosome 3q (Harris et al., 1995). Iron export from certain tissues is disrupted in these patients because ceruloplasmin normally facilitates iron export by converting it from Fe^2+^ to Fe^3+^, the form of iron that can be loaded onto transferrin. The retina, brain, and pancreas become iron overloaded in patients with aceruloplasminemia. Clinically, aceruloplasminemia includes the triad of retinal degeneration, dementia, and diabetes (Yamaguchi et al., 1998). The retinal findings in aceruloplasminemia have only been reported in a few patients. All of them have showed retinal pigmentary abnormalities, and one had subretinal macular deposits similar to drusen (Dunaief et al., 2005). Fluorescein angiography (FA) showed RPE atrophy resulting in window defects in the macula. The drusen-like opacities seen by ophthalmoscopy caused blocked fluorescence on the FA. The drusen-like deposits were first observed at age 47 and became smaller and spread out in a centrifugal fashion over the subsequent 9 years. Interestingly, the patient had yellow pingueculae in both eyes and Perls' Prussian blue staining revealed iron overloaded epithelial cells in the pinguecula. Histopathology of this case revealed RPE iron accumulation, hypertrophy, hyper and hypo-pigmentation, autofluorescence, sub-RPE and sub-retinal drusen.

Similarly, histopathology in mice with combined deficiencies of Cp and Heph has been studied (Hahn et al., 2004b). By 6–9 months, these mice demonstrated RPE iron accumulation and hypertrophy. Focal RPE hyperplasia and necrosis, local photoreceptor loss, and subretinal neovascularization were also observed in some mice. Electron microscopy of hypertrophic RPE showed increased phagosomes, lysosomes, and sub-RPE deposits of wide-spaced collagen, resembling some hallmarks of AMD (Figure 2).

### Hemochromatosis

There are two types of hemochromatosis: primary and secondary. As a hereditary disease, primary hemochromatosis is characterized by excessive iron accumulation in the heart, pancreas, liver, and other tissues (Pietrangelo, 2006). Clinically, this results in cardiomyopathy, diabetes, cirrhosis, arthritis, testicular failure, and darkening of the skin. Several mutations can lead to hereditary hemochromatosis. The most common mutation resides in the histocompatibility leukocyte antigen class I-like protein involved in iron homeostasis (HFE) gene product (Feder et al., 1998). This protein normally binds the TfR and forms a stable complex, thus lowering the affinity of the receptor for transferrin. The mutation affects complex formation, resulting in more transferrin binding to TfR and subsequently more iron import into the cell. HFE also regulates the expression of Hepc. Another genetic form of hereditary hemochromatosis results from mutation in ferroportin, reducing iron export from cells (Pietrangelo, 2004). Hereditary hemochromatosis can also result from mutations in Hepc, hemojuvelin (responsible for juvenile hemochromatosis), or TfR 2 (Pietrangelo, 2005; Nemeth and Ganz, 2006). In three cases with hereditary hemochromatosis, retinal abnormalities include drusen and iron in the peripapillary RPE, ciliary epithelium, and sclera (Roth and Foos, 1972).

Evidence from mice supports the possibility that primary hemochromatosis may result in retinal iron accumulation and degeneration. The mouse retina expresses HFE and HJV (Martin et al., 2006; Gnana-Prakasam et al., 2009b), and in knockouts of each of these genes, the retina accumulates iron and exhibits degeneration (Gnana-Prakasam et al., 2009a, 2012). In addition, the RPE exhibits a proliferative phenotype in mice lacking these genes.

Secondary hemochromatosis, or acquired hemochromatosis, results from iron intake during the process of multiple transfusions that are administered to patients with sickle cell anemia and thalassemia. Defects in Bruch's membrane underlying the RPE, called angioid streaks, can also be observed in these patients. However, it is difficult to differentiate the effects of iron overload, intraocular hemorrhage, and chelation therapy on the retina. It is possible that these patients have retinal iron overload. Alternatively, if local control of iron homeostasis in the retina prevents excess iron accumulation despite systemic iron overload, retinal iron level may not be affected. Retinal iron quantification and localization in *postmortem* eyes will help to address these issues.

### Friedreich's ataxia

Friedreich's ataxia (FRDA) is an autosomal recessive neurodegenerative disease characterized by progressive ataxia. In most patients with FRDA, a gene encoding the mitochondrial protein frataxin contains a mutation that leads to progressive iron accumulation in mitochondria. Most patients with Friedreich's ataxia can develop a late-onset progressive optic neuropathy, indicating ganglion cell involvement (Porter et al., 2007). A published case in a 59-year-old woman with FRDA documented severe optic neuropathy with rapid-onset catastrophic vision impairment (Porter et al., 2007). Color fundus photos showed a pale optic disc and scattered fleck-like yellow deposits throughout the macula. These yellowish deposits were auto-fluorescent, suggesting the presence of lipofuscin. Additionally, a pigmentary retinopathy has also been associated with FRDA.

### Pantothenate kinase-associated neurodegeneration (PKAN)

Pantothenate kinase-associated neurodegeneration (PKAN), an autosomal recessive neurodegenerative disorder which used to known as the Halervorden-Spatz syndrome, is characterized by iron accumulation in the brain resulting in dystonia, choreoathetosis, rigidity, spasticity, tremor, dementia, or psychomotor retardation (Koeppen and Dickson, 2001). The disease is due to mutations in the pantothenate kinase 2 gene which produces phosphopantothenate (Zhou et al., 2001). Phosphopantothenate condenses with cysteine in the production of coenzyme A. The mutation results in deficient phosphopantothenate and increased cysteine which binds iron. As a result, iron accumulates in areas of the brain normally containing the most iron, including the medial globus pallidus and subtantia nigra. In the retina where coenzyme A is in the highest demand, disrupted membrane biosynthesis is likely to occur. Rod photoreceptors are constantly shedding outer segments and synthesizing new membrane discs and deficit of CoA may explain the retinopathy resulting from PKAN. Clinical features of PKAN include childhood onset, dystonia or psychomotor retardation (Koeppen and Dickson, 2001). Retinal features in these patients include a pigmentary retinopathy with attenuated arterioles, foveal hyper-pigmentation, posterior pole depigmentation, bone spicules, bull's eye maculopathy, and flecked retina (Newell et al., 1979; Luckenbach et al., 1983). The flecks and macular annulus correlated to melanolipofuscin-contaning macrophages. Nearby RPE cells were hypertrophic with aggregated melanolipofuscin inside. The bone-spicule pattern seen by ophthalmoscopy corresponded to melanin pigment in RPE cells found around the retinal vasculature. Histology demonstrated total loss of photoreceptor outer and inner segments. Mice with a knockout of pantothenate kinase 2 also show photoreceptor degeneration (Kuo et al., 2005).

### Siderosis

Ocular siderosis is a sight-threatening condition resulting from deposition of iron in intraocular tissue that leads to oxidative damage from radicals generated by ferrous iron. The most common cause is an intraocular foreign body, but it can also due to an intraocular hemorrhage. Clinical manifestations of this disease include corneal iron deposition, iris heterochromia, pupillary mydriasis, failure of accommodation, anterior subcapsular cataract, or lens discoloration. If there is involvement of the trabecular meshwork and Schlemm's canal, secondary glaucoma can develop (Cibis et al., 1959; Talamo et al., 1985; Sneed, 1988). In the retina, RPE clumping and atrophy can occur as well as retinal arteriolar narrowing and retinal detachment. Initially, electroretinography (ERG) a- and b- wave amplitude may increase, but as the siderosis progresses and photoreceptors degenerate, there is gradual decrease in amplitude (Knave, 1969).

Animal models of siderosis have shown both histopathologic and functional abnormalities in the retina. After 10 days of solid iron foreign body insertion into rabbit vitreous, degeneration of the outer nuclear layer and RPE was observed (Declercq et al., 1977). Consistent with morphological change, ERG measurements also showed a reduction in both the a- and b-wave amplitudes under both scotopic and photopic conditions. Cibis et al. studied pathological specimens of patients who had ocular siderosis and hemosiderosis. Sequelae of intraocular iron overload include contraction bands in the vitreous body and inner surface of the retina, proliferation and obliteration of blood vessels, retinal detachment, and retinal degeneration (Cibis et al., 1959).

In ocular siderosis, iron accumulates in the eye because it has been introduced acutely. With aging and disease where iron transport is dysregulated, iron accumulation is slower and more chronic. Yet, whether the iron accumulation is acute or chronic, the photoreceptors and RPE are the cell types most susceptible to iron-induced damage or death.

### Subretinal hemorrhage

Subretinal hemorrhage in the macula may disrupt vision function in a number of diseases including AMD, myopic degeneration, angioid streaks, and ocular histoplasmosis. In a study of patients with intraretinal and subretinal hemorrhage, it was shown that the visual acuity loss depends on hemorrhage size and ability of the tissue to clear the blood (Gillies and Lahav, 1983).

The possible mechanisms for vision dysfunction caused by subretinal hemorrhage include direct iron toxicity to photoreceptors, iron toxicity to the RPE, isolation of the photoreceptors from the RPE, cell migration and proliferation in the subretinal space, or proliferation of a fibrovascular membrane (Gillies and Lahav, 1983). Autologous blood injection into the subretinal space of albino rats and rabbits resulted in progressive degeneration of the photoreceptor with edematous change, and iron accumulation in the RPE and photoreceptor outer segments (Glatt and Machemer, 1982). Deferoxamine, an iron chelator, has been shown to protect retina from toxicity caused by subretinal blood in these rats (Youssef et al., 2002). In a similar experimental model using rabbits, iron was identified with Perls' staining in RPE and photoreceptors, and triamcinolone reduces photoreceptor apoptosis (Bhisitkul et al., 2008). Oxyhemoglobin is believed to be a mediator responsible for the pathology of blood in the retina. *In vitro*, experiments with oxyhemoglobin demonstrated that, when elevated, led to lipid peroxidation in retinal tissues (Ito et al., 1995). The hemoglobin-binding protein hemopexin may protect retina from heme-mediated toxicity. RPE cells bind and internalize the heme–hemopexin complex from the retina and thus facilitate clearance of sub- or intra-retinal blood (Hunt et al., 1996).

## Potential therapeutics

Since iron-mediated oxidative damage may be involved in the pathogenesis of AMD, it is reasonable to assume antioxidants and iron chelators may reduce the incidence and progression of AMD. It has been shown by Age-Related Eye Disease Study (AREDS) that supplemental zinc, vitamin C, vitamin E, and β-carotene can reduce the risk of AMD progression, and it is likely that additional antioxidants may further protect or slow the AMD progression. Given that iron is one of the most potent sources of oxidative damage via hydroxyl radical production in the Fenton reaction, and since the antioxidants used in the AREDS study may not be able to eliminate all of the hydroxyl radical generated by iron, iron chelators may prove to be a useful complement to AREDS vitamins. Several reports indicate that iron chelation may be beneficial in neurological diseases such as Alzheimer's disease and Parkinson's disease, Huntington's disease and FRDA (Richardson, 2004; Zheng et al., 2005). It is plausible that iron chelation may also be effective in treating retinal disease associated with iron overload. Iron-binding proteins could also be therapeutically useful, as transferrin has been shown to protect against hereditary retinal degeneration in mice (Picard et al., 2010).

However, there are challenges with choosing clinically available iron chelators. Ideally, the iron chelator for retinal degeneration therapy should be absorbed easily in sufficient volume through the GI tract, and transit the blood–retinal barrier efficiently. Such chelators are likely to be uncharged, lipid soluble, and of small molecular size to facilitate passage through the blood–retinal barriers (Maxton et al., 1986; Kalinowski and Richardson, 2005). Additionally, the ideal chelator might exclusively bind iron but no other biologically important divalent metals such as Zn^2+^ (Liu and Hider, 2002).

Currently, the clinically available iron chelators include deferoxamine, deferiprone, and deferasirox. Another potentially therapeutic iron chelator is salicylaldehyde isonicotinoyl hydrazone (SIH). The advantages and disadvantages of each of these chelators are summarized by Mehta (Mehta and Dunaief, 2012). Previously, *in vitro* experiments demonstrated iron in the RPE and Bruch's membrane can be chelated with deferoxamine. A recent study (Obolensky et al., 2011) demonstrated that treatment with zinc-deferoxamine reduced retinal oxidative stress and enhanced photoreceptor survival, leading to both functional and structural rescue in the rd10 model of retinitis pigmentosa. However, deferoxamine is a cumbersome iron chelator requiring subcutaneous or intravenous administration due to its poor absorption by the GI system. In addition, deferoxamine has serious systemic side effects including pulmonary toxicity, bony changes, growth failure, and promotion of *Yersinia enterocolitica* infections (De Virgiliis et al., 1988; Brill et al., 1991; Tenenbein et al., 1992). Deferoxamine can also cause retinotoxicity. Haimovici et al. describe macular and peripheral pigmentary changes, as well as reduction in retinal function as indicated by reduction in ERG amplitude and electrooculogram light-peak to dark-trough ratios (Haimovici et al., 2002).

In contrast, deferiprone can be administered orally with fewer systemic side effects which can be prevented by careful monitoring. Oral deferiprone was found to be effective in decreasing retinal iron levels and oxidative stress in mice with age-dependent iron accumulation from combined Cp and Heph deficiency (Hadziahmetovic et al., 2011b). Unlike deferoxamine, deferiprone was not found to be toxic to the mouse retina. The iron chelator salicylaldehyde isonicotinoyl hydrazone was also found to reduce levels of reactive oxygen species and prevent RPE cell death in human RPE cell lines with exposure to oxidative stress (Kurz et al., 2009; Lukinova et al., 2009). In the experiments conducted by Lukinova et al., the RPE cells treated with SIH were also resistant to oxidative stress induced by staurosporine, anti-Fas, and exposure to A2E plus blue light (Lukinova et al., 2009). We also found that deferiprone can rescue light-damage-induced photoreceptor death in which iron dysregulation is not the primary cause of the degeneration (Song et al., 2012). Iron chelators, such as deferiprone, which have a lower affinity for iron than deferoxamine, may have less potential to cause side effects by removing iron from proteins that require an iron cofactor. Taken together, these results indicate that iron chelation could protect the retina against a broad range of insults. This has promising implications for the treatment of retinal diseases.

### Conflict of interest statement

The Dunaief labs receives research funding from ApoPharma, Inc., maker of deferiprone and Joshua L. Dunaief is listed an inventor in a patent pending for the use of deferiprone in retinal disease.
